# Extrusion of Sodium Hypochlorite in Oval-Shaped Canals: A Comparative Study of the Potential of Four Final Agitation Approaches Employing Agarose-Embedded Mandibular First Premolars

**DOI:** 10.3390/jcm13102748

**Published:** 2024-05-07

**Authors:** Aalisha Parkar, Kulvinder Singh Banga, Ajinkya M. Pawar, Alexander Maniangat Luke

**Affiliations:** 1 Department of Conservative Dentistry and Endodontics, Nair Hospital Dental College, Mumbai 400008, India; aalishaparkar17@gmail.com (A.P.); ksbanga@gmail.com (K.S.B.); 2 Department of Clinical Sciences, College of Dentistry, Ajman University, Al-Jruf, Ajman P.O. Box 346, United Arab Emirates; a.luke@ajman.ac.ae; 3 Centre of Medical and Bio-Allied Health Science Research (CMBHSR), Ajman University, Al-Jruf, Ajman P.O. Box 346, United Arab Emirates

**Keywords:** sodium hypochlorite extrusion, agitation techniques, apical extrusion, XP-Endo Finisher, root canal irrigation

## Abstract

**Background:** The aim of this investigation was to assess the apical extrusion potential of sodium hypochlorite (NaOCl) in agarose-embedded mandibular first premolars employing four final agitation procedures. **Methods:** Based on CBCT confirmation of single oval-shaped canals, one hundred extracted mandibular first premolars were chosen. Using 5.25% NaOCl, the teeth were prepared using the XP Endo Shaper and divided into experimental and control groups. The following were the experimental groups: Group 1 comprised the XP-Endo Finisher, Group 2 the Ultrasonic Activation, Group 3 the Gentle File Finisher Brush, and Group 4 the 27-gauge side-vented needle. To test extrusion, the teeth were placed in a 0.2% agarose gel that contained the pH-sensitive dye m-cresol purple, allowing pixel quantification via ImageJ software (version 1.54i). **Results:** The XP Endo Finisher featured the most pixels, depicting higher apical extrusion (*p* < 0.01), followed by the side-vented needle, Gentle File Finisher Brush, and PUI, while the Control Group endured no extrusion. **Conclusions:** The effective irrigation method for root canal therapy is critical, especially in situations of open apices, resorption, or perforation. According to in vitro experiments, the XP-Endo Finisher has the maximum sodium hypochlorite extrusion, which is determined by parameters such as apical preparation size and irrigation system.

## 1. Introduction

Numerous studies have indicated the significance of root canal debridement for the effectiveness of endodontic procedures [1,2,3]. Microorganisms must be eliminated from the intricate root canal system to prevent recurrent infections and preserve overall oral health. There are researchers who have emphasized the role of disinfection in reducing bacterial loads and creating a healing environment [4] and the need for thorough cleaning to prevent recurrence and guarantee long-term tooth preservation [5]. The importance of disinfection in avoiding apical periodontitis and its favorable association with adequate endodontic results are highlighted in the literature [6,7]. Additionally, Gomes et al. [8] stress the importance of disinfection in reducing the microbial load inside the root canal system and stopping the progression of infection to preserve the long-term health of the tooth.

Irrigant selection in root canal therapy is crucial, and sodium hypochlorite (NaOCl) has become essential because of its strong antibacterial qualities and effective disintegration of organic tissue [9,10,11]. NaOCl is well known for its capacity to effectively target a wide range of bacteria that are frequently seen in infected root canals [9,11,12], and its exceptional ability to maneuver through complex root canal morphology to difficult-to-reach sites for efficient disinfection is highlighted in the literature [13]. For successful endodontic irrigation, its ability to dissolve necrotic material is essential [9,12,14].

Although NaOCl is considered effective in root canal therapy, there are several drawbacks to its use, most notably the possibility of apical extrusion. Apical extrusion is defined as the accidental displacement of the irrigant beyond the apex of the tooth. It can cause harm to the periapical tissues and is linked to symptoms such as pain, swelling, and inflammation following endodontic treatment [15,16]; minimizing this phenomenon to maintain patient comfort and avoid consequences is vital. Furthermore, NaOCl extrusion has the potential to harm neighboring structures. This emphasizes the necessity of reducing this risk while undergoing root canal therapy. Apical extrusion must be minimized for the comfort of the patient as well as to reduce the possibility of complications that may jeopardize the root canal treatment’s overall effectiveness [17].

Oval-shaped root canals are complicated, and traditional nickel–titanium (NiTi) rotary tools frequently have limitations while treating such canals. This is because traditional rotary instrumentation usually prepares a round cross-sectional shape, which results in disproportionate dentin removal, with some regions receiving considerable dentin removal while others receive none. Research has indicated that the effectiveness of irrigation strategies in oval-shaped canals can differ based on the irrigant type, irrigation volume and pressure, and activation methodology [18,19,20]. Considering oval root canals have a larger surface area and a more irregular form, which might alter the flow and distribution of irrigation solutions, using oval-shaped teeth in a study on irrigation techniques is crucial. A better understanding can be gained of how well various irrigation strategies work to remove pathogens, debris, and necrotic tissue from difficult canals by using oval-shaped teeth in studies [18,19,20,21,22].

Numerous irrigation strategies in endodontic therapy, including conventional needle irrigation, passive ultrasonic irrigation, sonic irrigation, and negative apical pressure, have been thoroughly investigated to understand the apical extrusion risk associated with each irrigation strategy [21,22]. The goal of these assessments is to improve endodontic procedures with a focus on accuracy and patient safety. Nonetheless, it is important to remember that prior research frequently disregarded the influence on periapical tissues during extrusion evaluations, which may have led to an exaggerated appraisal of the issue and obscured its actual consequences for patient care. By providing a more accurate assessment of the extrusion, in vitro simulations enhance the security and quality of endodontic treatments [21,22]. Therefore, it is crucial to use a thorough methodology in apical extrusion research that takes periapical tissues into consideration.

In endodontic research, the modified unique closed-system agarose gel model offers a novel way to examine apical extrusion. This model allows for experiment integrity and variable control while providing a realistic assessment of extrusions. To directly compare the final irrigation cycle, the teeth in this model are instrumented prior to implantation, hence removing the instrumentation variable [23]. 

This study’s objective is to assess the efficacy of several agitation techniques, including the side-vented needle, Passive Ultrasonic Activation, Gentle File Finisher Brush, and XP Endo Finisher, in decreasing NaOCl apical extrusion. The goal of the project is to improve scientific rigor, minimize NaOCl extrusion, and contribute to the creation of evidence-based standards for endodontic operations that are safer and of better caliber.

## 2. Materials and Methods

### 2.1. Ethical Clearance

The Nair Hospital Dental College’s Institutional Ethics Committee in Mumbai, India granted clearance for the study (EC-210/CONS/ND 113/2023; approval date: 8 December 2023) in accordance with the Declaration of Helsinki.

### 2.2. Sample Size Estimation

G*Power 3 for Windows was used to calculate the sample size. The irrigant extrusion outcome of a previous study by Mitchell et al. [24] was used to estimate the sample size. For this investigation, the effect size was fixed at 1.20. The alpha-type error was 0.05, the beta power was 0.90, and the N_2_/N_1_ ratio was 1. It was estimated that 14 specimens would be required for the experimental and control groups; however, to reduce the chance of specimen loss, 20 teeth per group were used.

### 2.3. Specimen Selection

**Figure 1:**
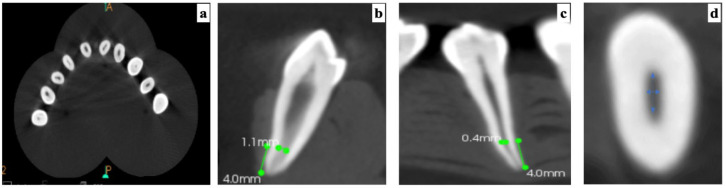
**Specimen selection:** (**a**) teeth placed into a wax mold for assessment of the presence of oval-shaped canals; (**b**) buccolingual dimension of the canal at 4 mm from the apex; (**c**) mesiodistal dimension of the canal at 4 mm from the apex; (**d**) buccolingual diameter 2.5 times the canal’s mesiodistal diameter.

A pilot investigation and power analysis at a power level of 90% revealed a minimum requirement of 19 teeth in each group. Following that, we collected 250 extracted mandibular first premolars, from which 100 teeth having oval-shaped canals confirmed through CBCT evaluation were selected. To analyze the number, curvature, and size of the canals in each tooth, digital radiographs were acquired from the buccal and proximal viewpoints. The teeth were then placed into wax molds with 3 cm spacing and 10 teeth per mold (Figure 1a).

The Kodak 3D CS 9300 system (Carestream, Atlanta, GA, USA) was used to perform cone-beam computed tomography (CBCT) on the teeth to identify the existence of a single oval-shaped canal. One hundred teeth with buccolingual dimensions (B/L) 2.5 times the canal’s mesiodistal dimensions (M/D), measuring 4 mm coronal to the apex, were selected (Figure 1b–d). All teeth were evaluated at 80 kVp and 2 mA with a 5.1 × 3.7 cm field of view and a voxel size of 300 μm.

Two expert observers reviewed the CBCT scans: an endodontist with 9 years of experience and an oral radiologist with 12 years of experience. To guarantee interobserver reliability, observations were calibrated using CS 3D imaging software (Carestream, Dental). In situations of disagreement, a third interpretation by an endodontist with 11 years of expertise was used to make the final decision.

The inclusion criteria were as follows:

1. The existence of a single oval-shaped canal;
2. A minimum working length (WL) of 22 mm;
3. Straight roots with less than 10% curvature according to the Schneider technique;
4. Fully developed apex;
5. No existence of cracks or apical resorption.

Teeth with an apical diameter greater than a #30k file and those that failed to satisfy the inclusion criteria were eliminated from the study.

### 2.4. Instrumentation

To reduce potential inconsistencies caused by the movement of agarose gel in the experimental setup, root canal preparation was performed prior to embedding in agarose gel. At 20× magnification (Zumax Medical Co., Ltd., Suzhou, China), each tooth was examined to make sure the apices had developed and that there were no fractures or root resorption. 

Following traditional means of accessing cavities with a No. 2 round bur and coronal flaring with No. 3 and No. 4 Gates Glidden burs, canal negotiation was initiated with a Mani K-File ISO #10 25 mm. Under 20× magnification, the file point became apparent at the apical foramen, confirming patency. The WL was reduced by 1.0 mm. To establish a glide path to the WL, a ProGlider (#13/0.02; 300 rpm and 2 N.cm) instrument (Dentsply-Maillefer) was used. Specimens were submerged in a warm water bath (37 ± 2 °C) to replicate conditions found in clinical settings.

An XP-endo Shaper single file (XPS, FKG Dentaire, Switzerland) was placed into the coronal third and triggered in slow up-and-down motions (800 rpm and 1 Ncm). The preparation time was estimated only while the file was spinning inside the canal. After attaining WL, five up-and-down motions were performed to produce a final preparation of 30/0.04, which was validated by fitting a 30/0.04 gutta-percha cone. All teeth received the same final apical preparation, which took into account both two-dimensional and three-dimensional quantitative characteristics.

During instrumentation, a 15 mL warm solution of 5.25% NaOCl was used to irrigate the canal. Following preparation, the teeth were soaked in 5% sodium thiosulfate for 10 min. Any remaining NaOCl was then neutralized with phosphate-buffered saline.

### 2.5. Embedding of the Samples

**Figure 2:**
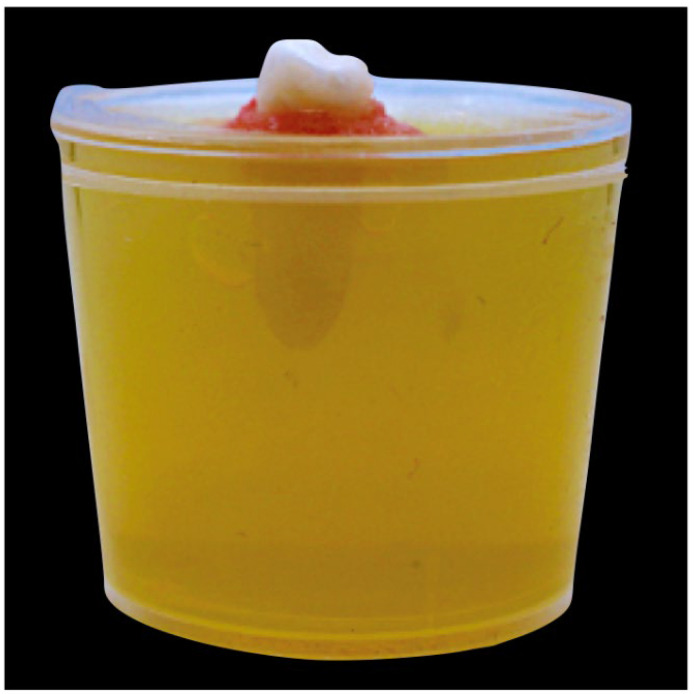
Prepared tooth mounted in agarose gel and fixed using self-curing acrylic resin.

A 0.2% agarose gel was prepared using TAE (Tris-acetate-EDTA) buffer solution in which 1 mL of 0.1% m-cresol purple (Lab Chem) was added. The experiment was carried out within 20 min of the gel setting. For each process, a 25 mL transparent plastic container served to lock the tooth in place with self-curing acrylic resin (DPI, India), preventing movement that may jeopardize uniformity (Figure 2). A 30/0.4 gutta-percha cone was used to prevent gel penetration into the apical foramen. The container was filled with 0.2% agarose gel (SRL) (pH 7.2–7.4) up to the cervical level of the crown. M-cresol purple changes color based on pH level, from yellow at pH 7.4 to purple at pH 9. A purple hue shift indicates that NaOCl (pH 11) is present in the gel. Pictures (B/L and M/D) were taken prior to the activation of the irrigant at a fixed distance of 30 cm with a fixed field of vision and other standardized conditions.

### 2.6. Irrigation Systems and Procedures

**Figure 3:**
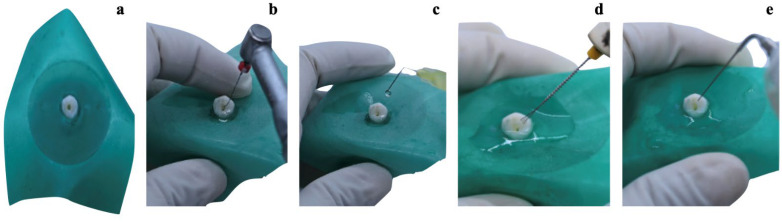
**Irrigation agitation/activation techniques:** (**a**) a gel model setup along with rubber dam, (**b**) the final agitation using an XP Endo Finisher, (**c**) the final agitation using a side-vented needle, (**d**) the final agitation using a Gentle File Finisher Brush, and (**e**) the final agitation using Passive Ultrasonic Activation.

This study examined the following irrigation approaches: the XP Endo Finisher (Group 1), Passive Ultrasonic Activation (Group 2), Gentle File Finisher Brush (Group 3), and side-vented needle (Group 4). A rubber dam and gingival barrier shielded the sole operator from the procedure and prevented him from ascertaining how much extrusion was made into the agarose gel in each step of the process (Figure 3a). All groups received 5.25% NaOCl irrigation. A pH meter was used before each procedure to validate the standard pH of 11.4 for NaOCl. The irrigation duration for all systems was set to 30 s, and the aggregate volume of irrigant for final agitation in all groups was 2 mL. In the Control Group, a 27-gauge side-vented needle was utilized to passively fill the pulp chamber area with NaOCl until it reached saturation; no further irrigant was added.

For the activated mechanisms, the device tip was placed into the root canal at 1 mm from the working length (WL) for the Gentle File Finisher Brush, side-vented needle, and Passive Ultrasonic Activation and at the WL for the XP Endo Finisher. In the XP Endo Finisher group, the device was inserted into the canal without being rotated. Once inserted, rotation (800 rpm and 1 Ncm) began, and the instrument was triggered with gradual and smooth movements of 7 to 8 mm along the long axis from the tooth to the WL (Figure 3b). The irrigation equipment was positioned passively to avoid contact with the canal walls during operation. In the side-vented needle group, the irrigant was agitated with 2 to 3 mm vertical strokes (Figure 3c). In the Gentle File Finisher Brush group, the device was rotated at 6500 rpm (Figure 3d). For the Passive Ultrasonic Activation group, the ultrasonic tip with a diameter of 20/0.2 was utilized for 30 s at an amplitude of 2 mm, 1 mm short of the actual working length, without impacting the canal walls (Figure 3e). Following each cycle, the leftover irrigant was aspirated from the canal, and the canal was dried with paper points.

### 2.7. Extrusion Evaluation

**Figure 4:**
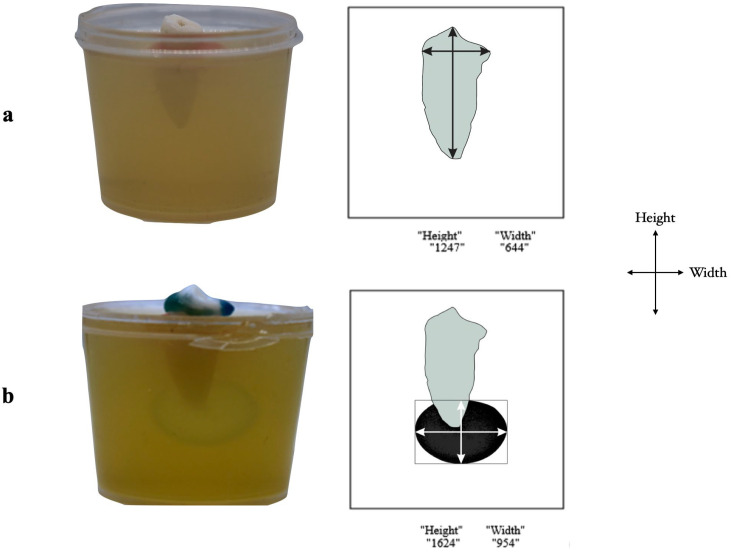
**Evaluation of the extruded hypochlorite:** (**a**) the preoperative image before irrigation initiation and its corresponding pixel and (**b**) the postoperative image showing the extruded NaOCl in the agarose gel model and its corresponding pixel.

The tooth/gel model was positioned in a light-box for transillumination and digitally photographed (Canon, 100 mm lens) in the buccal/lingual (BL) and mesial/distal orientations by using a camera placed at an exact distance (30 cm). To evaluate NaOCl extrusion into the gel, each sample was captured prior to the first irrigation period, visually inspected before subsequent irrigation periods, and then captured precisely 20 min after initiating the irrigation period. The pictures were processed using ImageJ software to calculate the region of the gel color shift expressed in pixels on the BL and MD views (Figure 4). The “purple” area’s perimeter was determined using a combination of methods. First, the most important factor was optical density, which measured the strength of the most noticeable pixel in a region. This offered a starting point for drawing the border. Secondly, the application of the compound fitting technique allows for the simultaneous fitting of neighboring clusters of spots. Computational efficiency was guaranteed by this approach, particularly when addressing overlaps between locations. Third, the definition of a “compound area” needed to be understood. By calculating the area that encompassed each spot in a compound, it was possible to enclose the extrusion and guarantee that the spots in one compound did not impact the spots in another. Lastly, locations whose pixel intensities were above a certain threshold were used to determine the purple line that marked the boundary. This all-inclusive method, which made good use of optical density, compound fitting, and intensity thresholds, guaranteed precise identification of the boundary of the “purple” area. Research by Karasu et al. [25] outlines the general notion of determining the percentage in their study. The pixel percentage was determined using the formula: preoperative pixel—postoperative pixel/preoperative pixel × 100.

### 2.8. Statistical Analysis

After performing the Shapiro–Wilk normality test, the data were normally distributed (*p* > 0.05). Descriptive statistics for the means and standard deviation were obtained. An analysis of variance (ANOVA) with a post hoc Tukey (HSD) test was performed to compare the groups’ extrusion values. The homogeneity of variance was calculated using Levene’s Test for Equality of Variances. The *p*-value for Levene’s Test was 0.749, which met the condition of variance homogeneity. All the statistical calculations were performed using Statistical Packages for Social Sciences (SPSS) IBM SPSS Statistics for Windows, Version 21.0. Armonk, New York: IBM Corp. A *p*-value < 0.05 was considered statistically significant at a 95% confidence interval.

## 3. Results

The mean pixel percentages for each experimental group are presented in Table 1. The pixel percentage was highest in Group 1, followed by Groups 4, 3, and 2. These differences in mean pixel percentages were found to be statistically significant (*p* = 0.000).

The difference in means by pairwise comparisons of the pixel percentage was not statistically significant for Groups 2 and 3 (*p* = 1.000); however, the differences in means between other groups were statistically significant (*p* = 0.000) (Table 2).

## 4. Discussion

The effectiveness of four different final agitation techniques was the primary interest of this study’s innovative examination into the extrusion of sodium hypochlorite (NaOCl) in oval-shaped canals. The teeth that meet the selection criteria are those whose cone-beam computed tomography (CBCT) verification of a single oval-shaped canal ensures that difficult anatomical situations that occur in practice are replicated. This work bridges a significant vacuum in the literature by concentrating on oval-shaped canals, which pose particular difficulties for conventional instrumentation approaches. Irrigation techniques are particularly successful in complicated root canal geometries. Additionally, the use of contemporary agitation methods like the XP Endo Finisher and Gentle File Finisher Brush provides a thorough analysis of modern methods for root canal irrigation. This work adds to the body of data supporting safer and more efficient endodontic operations by offering insightful information about the dynamics of NaOCl extrusion using a rigorous approach and sophisticated imaging tools.

The experimental groups and the Control Group were substantially distinct in mean apical extrusion, with Group 1 demonstrating the maximum extrusion, followed by Groups 4, 2, and 3, and finally the Control Group. The outcomes demonstrated statistically significant variations between the Control Group and the other experimental groups. Irrigant extrusion was represented by the mean pixel percentage, which differed dramatically between the groups. Group 1 had the highest percentage, followed by Groups 4, 3, and 2. Interestingly, the mean percentage of pixels between Groups 2 and 3 did not vary in a statistically significant manner, indicating that both groups were equally effective in reducing irrigant extrusion. However, significant variations were seen between Group 2 and the other experimental groups, as well as between Groups 3 and 4. These findings provide important information for well-informed decision making and better patient outcomes by highlighting the significance of choosing an effective final agitation strategy in root canal procedures to decrease apical irrigant extrusion.

Research provides strong evidence for the significant extrusion seen with the XP Endo Finisher [26,27,28]. This can be attributed to the XP Endo Finisher’s expanding motion and spoon-like shape at body temperature, which allows it to conform to the root canal architecture. The dynamic design creates a great deal of pressure and turbulence in the root canal environment, which makes deep penetration and effective debris removal possible [29]. The XP Endo Finisher’s greater apical reach may also have resulted in extrusion channel expansion, contributing to the highest extrusion levels [28].

A review of conclusions from multiple significant studies has clarified the noticeably reduced extrusion that can be detected with the side-vented needle. These studies have highlighted the advantageous lateral venting mechanism of the side-vented needle, which consists of a network of microchannels in the needle shaft. These channels actively guide irrigant flow outward, forming a cleaning cascade down the canal walls [30,31]. This design promotes effective debris removal, but it also involves some apical debris and irrigant displacement from the outward pressure imposed by the laterally dispersed irrigant, which functions similarly to a minor hydraulic ram [32]. Additionally, the side-vented needle’s narrow operative field and constrained design likely contribute to its lower total extrusion levels, in contrast to the XP Endo Finisher’s wider reach and expansive action [33].

Other researchers have presented compelling evidence that, in contrast to the XP Endo Finisher, passive ultrasonic irrigation (PUI) is the most effective method for reducing extrusion in endodontic procedures [34,35]. Because passive ultrasonic irrigation (PUI) may produce a high-velocity, low-volume irrigant jet that efficiently cleans the root canal system, it has been demonstrated to be beneficial in decreasing irrigant extrusion. While the modest volume of irrigant lowers the possibility of extrusion beyond the apical foramen, the high-velocity jet of irrigant produced by PUI can efficiently remove debris and disinfect the root canal system [34,35]. In particular, these mechanisms help to completely clear the canal lumen without producing significant apical pressure or causing severe fluid displacement [36,37,38,39]. Based on the significant current production caused by node creation along active files, PUI is efficient in decreasing irrigant extrusion. In comparison to traditional needle irrigation, this motion makes irrigation more successful since it lowers the possibility of pushing the irrigant into periapical tissue, which can happen when positive pressure is used. Furthermore, PUI breaks the apical vapor lock to allow lateral canal penetration, guaranteeing optimal irrigant penetration for the duration of the working length [40]. A number of variables, such as the depth of needle insertion, the degree of root canal curvature, the activation of NaOCl and its ongoing replenishment, and anatomic differences of the lateral canals, might affect how successful PUI is. The degree of irrigant penetration is also significantly influenced by other parameters, including power intensity, renewal rate, concentration, volume, flow rate, and activation time of the irrigant in the lateral canals [38,39]. The body of research demonstrates that PUI is a better approach for reducing extrusion and improving the safety profile of endodontic procedures.

The unique characteristics of the Gentle File Finisher Brush provide a thorough understanding of its much lower extrusion levels at rapid speeds [40]. The brush has six stainless steel strands that automatically extend outward when spun in a handpiece at 6500 rpm. Research by Khaleefah and Abdul emphasized the primary activity of the brush within the coronal canal and supported the belief that the GF brush showed a less direct apical effect. The agitation pressure probably plays a role in efficiently forcing irrigants and debris up against the canal walls, which limits net apical flow [41]. Because of its gentler agitation method, the Gentle File Finisher Brush achieves efficient cleaning while maintaining low extrusion levels [41]. This summary of findings, with an emphasis on the brush’s capacity to achieve a fine balance between safety and efficacy, offers strong support for the brush’s crucial role in endodontic process optimization.

Multifaceted comprehension acquired through several studies validates the use of agarose gel as our study’s paradigm. Prominent researchers lend credence to this decision [25,38]. Tanalp [38] and da Silva et al. [42] have emphasized the significance of adopting a model that closely mimics the clinical environment, which is consistent with our reasoning for using agarose gel because of its density approximation to human periapical tissue. The drawbacks of earlier models were highlighted by Yost et al. [43] especially those that concentrated on weight and electrolyte concentration factors, which our visually based gel model avoids. These studies validate that our model, which prioritizes volume over weight, not only avoids possible problems like NaOCl crystallization but also improves the experimental setup’s reliability, allowing for more accurate measurements of the intensity, orientation, and reach of the NaOCl extrusion. This multifaceted explanation supports our deliberate choice of agarose gel as a model in our experimental design and is based on a synthesis of the information from this research. According to previously performed studies [44], the quantity of extrusion may be precisely correlated with the evaluation of pixel number, and thus, it was utilized for the assessment of extrusion.

Even though the quantitative features of our study were the main emphasis, it is important to recognize the limitations. Despite efforts to standardize the process, the experimental model does not account for the existence of pulpal tissue or periapical tissues, which may function as obstacles to extrusion in vivo. An exact measurement of the density of periodontium cannot be predicted due to the loss of tissues in the periapical area produced by periapical lesions of varying proportions. In clinical circumstances, granulomatous or periradicular tissues encircle the tooth’s apex, perhaps restricting apical extrusion to some degree. As a result, attention was focused in this study to guarantee that the sample’s preoperative morphologic traits were distributed evenly throughout the groups. As the operator’s tactile skills were deemed more important, root canal preparation was conducted by an operator with proficiency in each of the performed steps.

Our study’s possible shortcomings include the fact that it did not assess the cleaning performance of each irrigation method within the root canal, as this was not the major emphasis. Also, the canal volume and taper were not considered in the current study. Furthermore, the density of the gel cannot be readily correlated with that of an intact periodontium or a periapical lesion, as a consistent density is not predicted. Additionally, qualitative characteristics such as the antigenic potential of the extruded material and its impact on host defense systems were not examined in this study. As a result, it is possible that the measured numerical values of the extruded irrigant may not accurately reflect clinical expectations.

## 5. Conclusions

Within the constraints of the conducted study, the XP Endo Finisher approach produced the maximum NaOCl extrusion, followed by the side-vented needle technique. Extrusion was the lowest in the Gentle File Finisher Brush and PUI methods, and the least in the Control Group. Final agitation procedures must be carefully chosen to reduce NaOCl apical extrusion and related dangers during endodontic therapy. More studies are needed to validate and advise endodontic practitioners, in particular in cases of resorption, perforation abnormalities, or roots that are young and have open apices.

## Figures and Tables

**Table 1:** Comparison of the mean pixel percentages of apical extrusion between groups.

Groups	Irrigation Device	N	Mean	Std. Dev.	F	*p*-Value
1	XP Endo Finisher	20	69.1146	3.32050	48.545	0.000
2	Side-vented needle	20	39.7493	11.21137		
3	Gentle File	20	39.8190	9.11926		
4	Passive Ultrasonic	20	52.5144	9.93773		

**Table 2:** Multiple comparisons of the mean pixel percentages of apical extrusion between groups 1, 2, 3, and 4.

(I) Groups	(J) Groups	Mean Difference (I-J)	*p* Value	95% Confidence Interval	
				Lower Bound	Upper Bound
Group 1	Group 2	29.36527	0.000	21.9514	36.7792
	Group 3	29.29558	0.000	21.8817	36.7095
	Group 4	16.60021	0.000	9.1863	24.0141
Group 2	Group 3	−0.06969	1.000	−7.4836	7.3442
	Group 4	−12.76506	0.000	−20.1790	−5.3512
Group 3	Group 4	−12.69538	0.000	−20.1093	−5.2815

## Data Availability

The data are available on request to the corresponding author.
